# Synthesis and crystal structure of *anti*-10-butyl-10,11,22,23-tetra­hydro-9*H*,21*H*-5,8:15,12-bis(metheno)[1,5,11]tri­aza­cyclo­hexa­decino[1,16-*a*:5,6-*a*′]di­indole

**DOI:** 10.1107/S2056989022003383

**Published:** 2022-04-05

**Authors:** Koji Kubono, Keita Tani, Yukiyasu Kashiwagi, Fumito Tani, Taisuke Matsumoto

**Affiliations:** a Osaka Kyoiku University, 4-698-1 Asahigaoka, Kashiwara, Osaka 582-8582, Japan; b Osaka Research Institute of Industrial Science and Technology, 1-6-50 Morinomiya, Joto-ku, Osaka 536-8553, Japan; c Institute for Materials Chemistry and Engineering, Kyushu University, 744 Motooka, Nishi-ku, Fukuoka 819-0395, Japan; d Institute for Materials Chemistry and Engineering, Kyushu University, 6-1 Kasuga koen, Kasuga, Fukuoka 816-8580, Japan

**Keywords:** crystal structure, carbazolophane, racemate, C—H⋯N hydrogen bonds, C—H⋯π inter­actions

## Abstract

The title compound, C_33_H_33_N_3_, is a carbazolophane, which is a cyclo­phane composed of two carbazole fragments. It has a planar chirality but crystallizes as a racemate in the space group *P*  . The mol­ecule adopts an *anti*-configuration, in which two carbazole fragments are partially overlapped. Both carbazole ring systems are slightly bent, with the C atoms at 3-positions showing the largest deviations from the mean planes. The dihedral angle between two carbazole fragments is 5.19 (3)°, allowing an intra­molecular slipped π–π inter­action [*Cg*⋯*Cg* = 3.2514 (8) Å]. In the crystal, the mol­ecules are linked *via* inter­molecular C—H⋯N hydrogen bonds and C—H⋯π inter­actions into a network sheet parallel to the *ab* plane. The mol­ecules of different sheets form other C—H⋯π inter­actions, thus forming a three-dimensional network.

## Abstract 2

In the title compound, the mol­ecule adopts an *anti*-configuration, in which two partially overlapped carbazole fragments form an intra­molecular slipped π–π inter­action. In the crystal, the mol­ecules are cross-linked *via* inter­molecular C—H⋯N hydrogen bonds and C—H⋯π inter­actions into a three-dimensional network.

## 1. Chemical context

Carbazole is characterized not only as a mol­ecule with electron-donating properties, but also as an emissive heteroaromatic chromophore (Wex *et al.*, 2017 ▸), so carbazole derivatives have attracted much attention for the construction of photofunctional devices such as solar cells (Gratia *et al.*, 2015 ▸) and organic light-emitting diodes (Kaji *et al.*, 2015 ▸). Poly(*N*-vinyl­carbazole) is a widely used photoconductive aromatic polymer, in which the formation of two types of excimers, partially overlapped (PO) and fully overlapped (FO) (sandwich) ones, was proposed (Sakai *et al.*, 1996 ▸). Our group has reported various carbazolophanes, which are cyclo­phanes composed of two carbazole fragments, as the models of excimers. Among these, aza-bridged carbazolophanes synthesized so far by cyclization reaction are limited to cyanamide bridg­ing (Tani *et al.*, 2001 ▸, 2007 ▸) and *o*-nitro­phenyl­sulfonamide bridging (Tani *et al.*, 2020 ▸). These bridges act as polar functional groups with the resonance effect. In the aza-bridged carbazolophanes, the PO and FO isomers have been isolated and their distinct difference in fluorescence spectra provided the evidence for existence of two types of excimers (Tani *et al.*, 2001 ▸; Ohkita *et al.*, 2002 ▸). Recently, optical resolution of aza-bridged PO carbazolophanes and their chiroptical properties, including circularly polarized luminescence, were reported (Tani *et al.*, 2020 ▸). However, the solubility of the aza-bridged carbazolophanes in common organic solvents is low, leading to difficulties in examining the solvent effect and in manufacturing photofunctional devices. Therefore, the title compound with an *N*-linear alkyl group, *anti*-10-butyl-10,11,22,23-tetra­hydro-9*H*,21*H*-5,8:15,12-bis(meth­eno)[1,5,11]tri­aza­cyclo­hexa­dec­ino[1,16-a:5,6-a′]diindole (cyclo­phane nomenclature: *anti*-3-but­yl-1^9^ *H*,5^9^ *H*-3-aza-1,5(3,9)-dicarbazola­cyclo­octa­phane), having good solubility in organic solvents, is a promising candidate for investigation of the photophysical and chiroptical properties of the carbazole chromophore. Previously, our group reported the EPR spectrum of the title compound, but no other chemical properties were examined because of the very low yield (Saiful *et al.*, 2006 ▸). Here, the modified synthesis and crystal structure of the title compound are reported. 
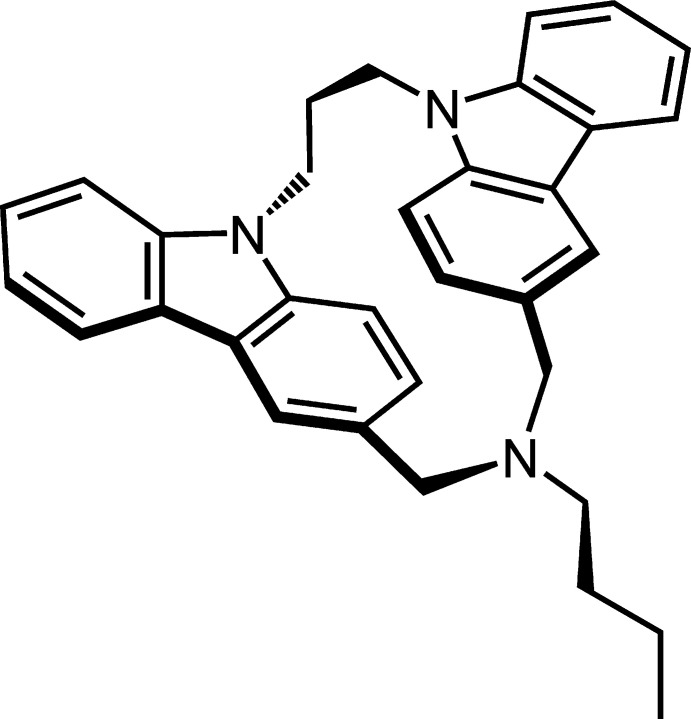


## 2. Structural commentary

**Figure 1:**
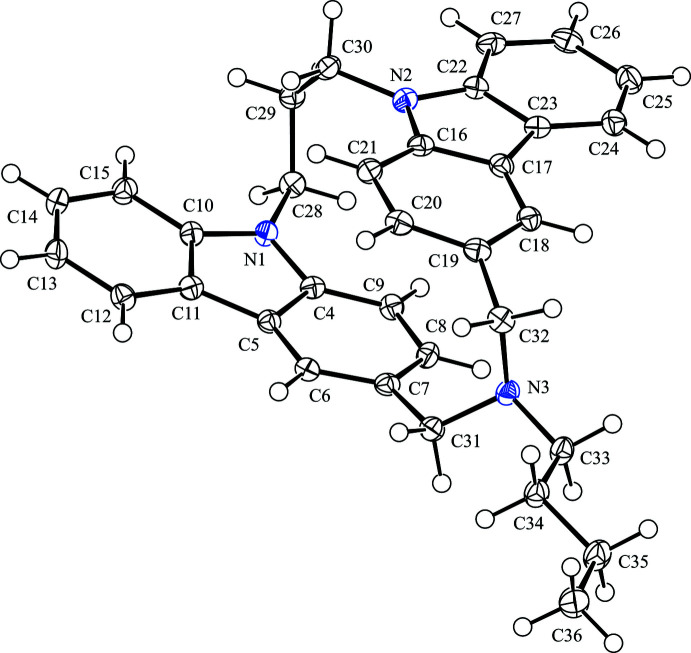
The mol­ecular structure of the title compound with the atom labelling. Displacement ellipsoids are drawn at the 50% probability level. H atoms are represented by spheres of arbitrary radius.

The title compound has a planar chirality but crystallizes as a racemate in the centrosymmetric space group *P* 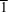 . The mol­ecular structure of the title compound is shown in Fig. 1 ▸. The mol­ecule adopts an *anti*-configuration, in which two carbazole fragments are partially overlapped with parallel orientation. The two carbazole fragments are slightly bent, with r.m.s. deviations of 0.064 (1) Å for the N1/C4–C15 ring system and 0.062 (1)Å for N2/C16–C27 ring system. In both carbazole fragments, the C atoms at the 3-positions bridged through the di­methyl­ene­amino group show the largest deviations from the mean planes [0.1177 (14) Å for C7 and −0.1082 (14) Å for C19]. The dihedral angle formed by two carbazole fragments is 5.19 (3)°, providing an intra­molecular slipped parallel π–π inter­action [*Cg*2⋯*Cg*5 = 3.2514 (8) Å; *Cg*2 and *Cg*5 are the centroids of the C4–C9 and C16–C21 rings, respectively; inter-planar distance = 3.0856 (6) Å; slippage = 1.099 Å]. In comparison, in the related PO compounds, the dihedral angles between two carbazole fragments and the centroid–centroid distances are 5.96 (6)° and 3.294 (4) Å for *N*-cyanamide-bridged [3.3](3,9)carbazolophane (BACKOG; Tani *et al.*, 2001 ▸), and 1.28 (7)° and 3.3259 (16) Å for *N*-*o*-nitro­phenyl­sulfonamide-bridged [3.3](3,9)carbazolophane (YUKYEL; Tani *et al.*, 2020 ▸). The N1⋯N2 distance between the N atoms at the 9-positions of the carbazole ring systems is 3.3776 (17) Å, slightly shorter than those in the above-mentioned related compounds [3.414 (4) and 3.461 (4) Å for the cyanamide-bridged and *o*-nitro­phenyl­sulfonamide-bridged carbazolophanes, respectively]. The bond angle C31—N3—C32 is 114.56 (11)°, smaller than those in the related compounds [119.9 (2) and 119.0 (3)° for the cyanamide-bridged and *o*-nitro­phenyl­sulfonamide-bridged carbazolophanes, respectively], that is, the hybridization of N3 atom is closer to *sp* ^3^ than to *sp* ^2^, reflecting the difference in the resonance effect of the substituent at the N3 atom.

## 3. Supra­molecular features

**Figure 2:**
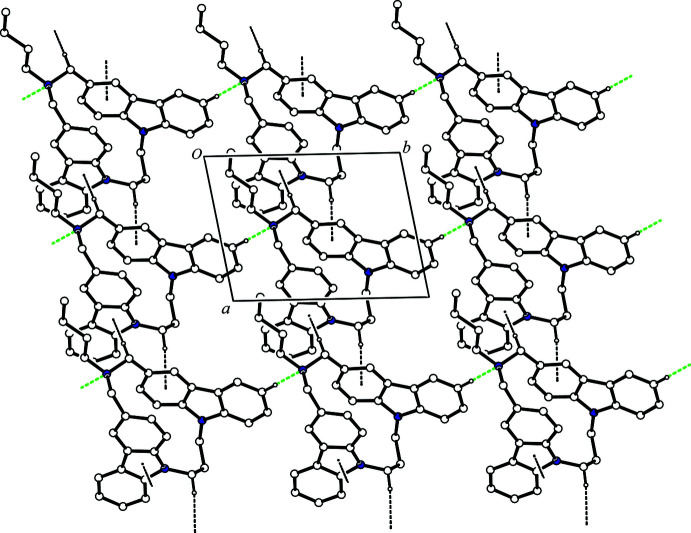
A packing diagram of the title compound viewed along the *c* axis, showing the two-dimensional network. The C—H⋯N hydrogen bonds and C—H⋯π inter­actions are shown as green and black dashed lines, respectively. H atoms not involved in these inter­actions have been omitted for clarity.

**Figure 3:**
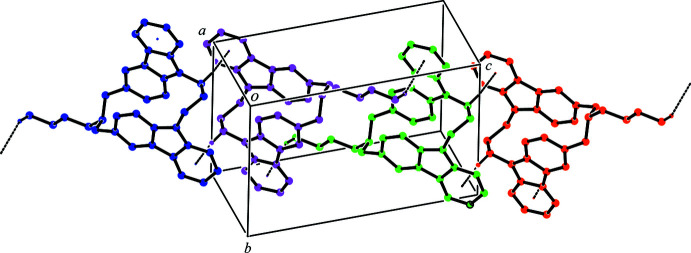
An excerpt of the crystal packing of the title compound showing the ribbon structure along [ 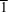 01]. The C—H⋯π inter­actions are shown as black dashed lines. H atoms not involved in these inter­actions have been omitted for clarity.

In the crystal, mol­ecules are linked by inter­molecular C—H⋯N hydrogen bonds (Fig. 2 ▸, Table 1 ▸), forming a *C*(9) chain motif running parallel to the *b* axis. The mol­ecules are further joined into columns along the *a*-axis direction by pairs of C—H⋯π inter­actions (C30—H30*A*⋯*Cg*2^iii^ and C31—H31*B*⋯*Cg*3^iv^; *Cg*2 and *Cg*3 are the centroids of the C4–C9 and N2/C16/C17/C23/C22 rings, respectively; see Fig. 2 ▸ and Table 1 ▸), thus network sheets parallel to the *ab* plane are observed (Fig. 2 ▸). Besides this, the mol­ecules belonging to different sheets are associated *via* a pair of C—H⋯π inter­actions (C28—H28*A*⋯*Cg*1^ii^; *Cg*1 is the centroid of the C22–C27 ring), forming a centrosymmetric dimer (Fig. 3 ▸). Another pair of C—H⋯π inter­actions (C36—H36*B*⋯*Cg*4^v^; *Cg*4 is the centroid of the C10–C15 ring) forms another centrosymmetric dimer (Fig. 3 ▸). As a result, a ribbon structure along [ 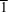 01] is formed (Fig. 3 ▸). Overall, the mol­ecules are cross-linked *via* inter­molecular C—H⋯N hydrogen bonds and C—H⋯π inter­actions into a three-dimensional network.

## 4. Database survey

A search of the Cambridge Structural Database (CSD, Version 5.42; May 2021; Groom *et al.*, 2016 ▸) using *ConQuest* (Bruno *et al.*, 2002 ▸) for compounds containing a carbazole skeleton gave 4473 hits, and for those containing two 3,9-di­methyl­enecarbazole fragments gave 49 hits. Among those, a search for the carbazolophane skeleton gave seven hits. Of these seven compounds, three structures are [3.3](3,9) carbazolophanes, two being PO [3.3](3,9)carbazolophanes with the same skeleton as in the title compound: *anti*-10-(2-nitro­benzen-1-sulfon­yl)-10,11,22,23-tetra­hydro-9*H*,21*H*-5,8:15,12-bis­(metheno)[1,5,11]tri­aza­cyclo­hexa­decino[1,16-*a*:5,6-*a*′]-di­indole (*N*-cyanamide-bridged PO [3.3](3,9)carbazolophane, YUKYEL; Tani *et al.*, 2020 ▸) and *anti*-3-cyano-3-aza-1(9,3),3(3,9)-dicarbazola­cycloocta­phane (*N*-*o*-nitro­phenyl­sulfonamide-bridged PO [3.3](3,9)carbazolophane, BACKOG; Tani *et al.*, 2001 ▸). One structure is *N*-cyanamide-bridged FO [3.3](3,9)carbazolophane, *syn*-3-cyano-3-aza-1(9,3),3(3,9)-dicarbazola­cyclo­octa­phane benzene clathrate (BACKIA; Tani *et al.*, 2001 ▸), in which the dihedral angle between two carbazole rings is 8.48 (10)°, and intra­molecular *Cg*⋯*Cg* distances are 3.322 (3) Å for the benzene rings bridged by cyanamide, 3.447 (2) Å for the central pyrrole rings and 3.792 (3) Å for the outer benzene rings. Three of the remaining four structures are PO [*m.n*](3,9)carbazolophanes; *anti*-ethenylene and 1,3-xylylene-bridged [2.5](3,9)carbazolophane (VELKON; Kumar *et al.*, 2006 ▸), *anti*-*N*-cyanamide-bridged [3.4](3,9)carbazolophane (KEYVAM; Tani *et al.*, 2007 ▸) and *anti*-*O*-oxa-bridged [3.5](3,9)carbazolophane (KEYVEG; Tani *et al.*, 2007 ▸). In these structures, the dihedral angles between two carbazole ring systems and intra­molecular *Cg*⋯*Cg* distances in the partially overlapped benzene rings are 31.69 (6)° and 3.8062 (15) Å for ethenylene and 1,3-xylylene-bridged [2.5](3,9)carbazolophane; 15.04 (9)° and 3.732 (3) Å for cyanamide-bridged [3.4](3,9)carbazolophane; 24.87 (11)° and 3.901 (3) Å (the average value of two independent mol­ecules) for oxa-bridged [3.5](3,9)carbazolophane. The last of the seven compounds is a FO carbazolophane, *syn*-cyclo­butane-bridged [2.4](3,9)carbazolophane (GOZGUX; Nakamura *et al.*, 1999 ▸) in which the dihedral angle between its carbazole fragments is 18.9 (2)°, and intra­molecular *Cg*⋯*Cg* distances are 3.517 Å for the benzene rings bridged by cyanamide, 4.167 Å for the central pyrrole rings and 4.242 Å for the outer benzene rings.

## 5. Synthesis and crystallization

A solution of 9,9′-(1,3-propanedi­yl)bis­[3-(bromo­meth­yl)-9*H-*carbazole] (560 mg, 1.00 mmol; Tani, *et al.*, 2001 ▸) in di­chloro­methane (100 mL) was added to a 500 mL flask, which contained a mixture of tetra­butyl­ammonium iodide (70.6 mg, 0.191 mmol) and *n*-butyl­amine (220 mg, 3.01 mmol) in di­chloro­methane (150 mL) and sodium hydroxide (1.00 g, 0.25 mol) in water (10 mL). Then, the flask was filled with argon and was stirred at room temperature for 3 d. The reaction mixtures were washed with water, then dried over anhydrous sodium sulfate. Solvent was removed under reduced pressure, and the residue was purified by silica gel chromatography (Wako-gel C-200, 10 g). Elution from hexa­ne:ethyl acetate (19:1) gave a white solid (41.5 mg, 9%). Elution from hexa­ne:ethyl acetate (10:1) gave mixtures including a *syn*-configuration (FO isomer), but they were difficult to separate. A part of the title compound was recrystallized from di­chloro­methane:ethanol (1:3) to give a colorless crystal suitable for X-ray diffraction. Melting point: 482–484 K. ^1^H NMR (CDCl_3_, 400 MHz) *δ* = 1.08 (*t*, *J* = 7.6 Hz, 3H), 1.57–1.60 (*m*, 2H),1.78 (*quint*, *J* = 7.6 Hz, 2H), 2.81–2.97 (*m*, 4H), 3.74–3.90 (*m*, 6H), 4.10–4.17 (*m*, 2H), 5.34 (*d*, *J* = 8.1 Hz, 2H), 6.38 (*br*, 2H), 7.25-7.30 (*m*, 2H), 7.46–7.53 (*m*, 4H), 7.67 (*s*, 2H), 8.11 (*d*, *J* = 7.6 Hz, 2H).

## 6. Refinement

Crystal data, data collection and structure refinement details are summarized in Table 2 ▸. C-bound H atoms were placed in geometrically calculated positions (C—H = 0.95–0.99 Å) and refined as riding with *U*_iso_(H) = 1.2*U*_eq_(C).

## Supplementary Material

Crystal structure: contains datablock(s) global, I. DOI: 10.1107/S2056989022003383/yk2167sup1.cif

Structure factors: contains datablock(s) I. DOI: 10.1107/S2056989022003383/yk2167Isup2.hkl

CCDC reference: 2161895

Additional supporting information:  crystallographic information; 3D view; checkCIF report

## Figures and Tables

**Table 1:** Hydrogen-bond geometry (Å, °) *Cg*1, *Cg*2, *Cg*3 and *Cg*4 are the centroids of the C22–C27, C4–C9, N2/C16/C17/C22/C23 and C10–C15 rings, respectively.

*D*—H⋯*A*	*D*—H	H⋯*A*	*D*⋯*A*	*D*—H⋯*A*
C13—H13⋯N3^i^	0.95	2.56	3.4858 (18)	166
C28—H28*A*⋯*Cg*1^ii^	0.99	2.72	3.4144 (15)	128
C30—H30*A*⋯*Cg*2^iii^	0.99	2.62	3.5224 (16)	152
C31—H31*B*⋯*Cg*3^iv^	0.99	2.83	3.7245 (15)	150
C36—H36*B*⋯*Cg*4^v^	0.98	2.99	3.9151 (18)	159

Symmetry codes: (i) 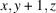 ; (ii) 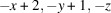 ; (iii) 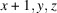 ; (iv) 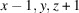 ; (v) 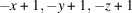 .

**Table 2:** Experimental details

Crystal data	
Chemical formula	C_33_H_33_N_3_
*M*_r_	471.64
Crystal system, space group	Triclinic, *P* 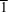
Temperature (K)	100
*a*, *b*, *c* (Å)	7.9106 (3), 10.4468 (4), 15.5735 (5)
α, β, γ (°)	79.988 (3), 77.717 (3), 77.909 (3)
*V* (Å^3^)	1218.33 (8)
*Z*	2
Radiation type	Cu *K*α
μ (mm^−1^)	0.58
Crystal size (mm)	0.40 × 0.13 × 0.05

Data collection	
Diffractometer	Rigaku XtaLAB Synergy
Absorption correction	Gaussian (*CrysAlis PRO*; Rigaku OD, 2022 ▸)
*T*_min_, *T*_max_	0.451, 0.972
No. of measured, independent and observed [*F* ^2^ > 2.0σ(*F* ^2^)] reflections	14067, 4827, 4383
*R*_int_	0.028
(sin θ/λ)_max_ (Å^−1^)	0.628

Refinement	
*R*[*F* ^2^ > 2σ(*F* ^2^)], *wR*(*F* ^2^), *S*	0.044, 0.118, 1.05
No. of reflections	4827
No. of parameters	326
H-atom treatment	H-atom parameters constrained
Δρ_max_, Δρ_min_ (e Å^−3^)	0.48, −0.31

Computer programs: *CrysAlis PRO* (Rigaku OD, 2022 ▸), *SHELXT2018/2* (Sheldrick, 2015*a* ▸), *SHELXL2018/3* (Sheldrick, 2015*b* ▸) and *CrystalStructure* (Rigaku, 2019 ▸).

## supplementary crystallographic information

### Crystal data

**Table:**

C_33_H_33_N_3_	*Z* = 2
*M**_r_* = 471.64	*F*(000) = 504.00
Triclinic, *P*1	*D*_x_ = 1.286 Mg m^−^^3^
*a* = 7.9106 (3) Å	Cu *K*α radiation, λ = 1.54184 Å
*b* = 10.4468 (4) Å	Cell parameters from 9429 reflections
*c* = 15.5735 (5) Å	θ = 2.9–74.8°
α = 79.988 (3)°	µ = 0.58 mm^−^^1^
β = 77.717 (3)°	*T* = 100 K
γ = 77.909 (3)°	Prism, colourless
*V* = 1218.33 (8) Å^3^	0.40 × 0.13 × 0.05 mm

### Data collection

**Table:**

Rigaku XtaLAB Synergy diffractometer	4383 reflections with *F*^2^ > 2.0σ(*F*^2^)
Detector resolution: 10.000 pixels mm^-1^	*R*_int_ = 0.028
ω scans	θ_max_ = 75.4°, θ_min_ = 2.9°
Absorption correction: gaussian (CrysAlisPro; Rigaku OD, 2022)	*h* = −9→9
*T*_min_ = 0.451, *T*_max_ = 0.972	*k* = −12→12
14067 measured reflections	*l* = −19→15
4827 independent reflections	

### Refinement

**Table:**

Refinement on *F*^2^	Secondary atom site location: difference Fourier map
*R*[*F*^2^ > 2σ(*F*^2^)] = 0.044	Hydrogen site location: inferred from neighbouring sites
*wR*(*F*^2^) = 0.118	H-atom parameters constrained
*S* = 1.05	*w* = 1/[σ^2^(*F*_o_^2^) + (0.057*P*)^2^ + 0.6261*P*] where *P* = (*F*_o_^2^ + 2*F*_c_^2^)/3
4827 reflections	(Δ/σ)_max_ < 0.001
326 parameters	Δρ_max_ = 0.48 e Å^−^^3^
0 restraints	Δρ_min_ = −0.31 e Å^−^^3^
Primary atom site location: structure-invariant direct methods	

### Special details

**Table:**

Geometry. All esds (except the esd in the dihedral angle between two l.s. planes) are estimated using the full covariance matrix. The cell esds are taken into account individually in the estimation of esds in distances, angles and torsion angles; correlations between esds in cell parameters are only used when they are defined by crystal symmetry. An approximate (isotropic) treatment of cell esds is used for estimating esds involving l.s. planes.
Refinement. Refinement was performed using all reflections. The weighted R-factor (wR) and goodness of fit (S) are based on F^2^. R-factor (gt) are based on F. The threshold expression of F^2^ > 2.0 sigma(F^2^) is used only for calculating R-factor (gt).

### Fractional atomic coordinates and isotropic or equivalent isotropic displacement parameters (Å^2^)

**Table:**

	*x*	*y*	*z*	*U*_iso_*/*U*_eq_	
N1	0.82658 (15)	0.71805 (11)	0.11798 (7)	0.0171 (2)	
N2	1.15258 (15)	0.47838 (11)	0.18124 (8)	0.0188 (2)	
N3	0.49011 (15)	0.28118 (11)	0.39375 (7)	0.0177 (2)	
C4	0.71367 (17)	0.63439 (13)	0.16544 (8)	0.0158 (3)	
C5	0.59504 (17)	0.69870 (13)	0.23257 (9)	0.0160 (3)	
C6	0.48501 (17)	0.62693 (13)	0.29675 (9)	0.0165 (3)	
H6	0.406279	0.668922	0.342939	0.020*	
C7	0.49126 (17)	0.49443 (13)	0.29271 (9)	0.0171 (3)	
C8	0.60034 (18)	0.43601 (13)	0.22033 (9)	0.0178 (3)	
H8	0.596903	0.347337	0.215192	0.021*	
C9	0.71166 (17)	0.50366 (13)	0.15691 (9)	0.0175 (3)	
H9	0.784875	0.462725	0.108815	0.021*	
C10	0.77834 (17)	0.83775 (13)	0.15155 (9)	0.0173 (3)	
C11	0.63514 (17)	0.82992 (13)	0.22327 (9)	0.0174 (3)	
C12	0.56582 (18)	0.93710 (13)	0.26950 (9)	0.0190 (3)	
H12	0.470434	0.932256	0.317992	0.023*	
C13	0.63778 (19)	1.05200 (14)	0.24394 (10)	0.0222 (3)	
H13	0.592392	1.125523	0.275587	0.027*	
C14	0.77841 (18)	1.05906 (13)	0.17086 (10)	0.0213 (3)	
H14	0.824434	1.138552	0.153186	0.026*	
C15	0.85017 (18)	0.95296 (14)	0.12471 (9)	0.0207 (3)	
H15	0.945449	0.958064	0.076184	0.025*	
C16	1.03369 (17)	0.44294 (13)	0.25712 (9)	0.0174 (3)	
C17	1.02490 (17)	0.30775 (13)	0.26373 (9)	0.0175 (3)	
C18	0.89825 (17)	0.25347 (13)	0.32861 (9)	0.0180 (3)	
H18	0.890067	0.163128	0.332509	0.022*	
C19	0.78436 (17)	0.33257 (14)	0.38739 (9)	0.0184 (3)	
C20	0.80863 (18)	0.46273 (14)	0.38464 (9)	0.0196 (3)	
H20	0.738393	0.513423	0.428779	0.024*	
C21	0.93065 (18)	0.52035 (14)	0.32029 (9)	0.0193 (3)	
H21	0.943592	0.609013	0.319239	0.023*	
C22	1.22754 (17)	0.36575 (13)	0.14118 (9)	0.0182 (3)	
C23	1.15213 (17)	0.25666 (13)	0.19060 (9)	0.0171 (3)	
C24	1.20764 (18)	0.13269 (14)	0.16252 (9)	0.0209 (3)	
H24	1.157845	0.059339	0.194712	0.025*	
C25	1.33597 (19)	0.11695 (14)	0.08733 (10)	0.0238 (3)	
H25	1.374559	0.032439	0.068095	0.029*	
C26	1.40972 (18)	0.22603 (15)	0.03911 (9)	0.0226 (3)	
H26	1.497276	0.213768	−0.012435	0.027*	
C27	1.35725 (18)	0.34962 (14)	0.06532 (9)	0.0211 (3)	
H27	1.407904	0.422325	0.032672	0.025*	
C28	0.97116 (17)	0.68287 (14)	0.04623 (9)	0.0186 (3)	
H28A	0.953335	0.746320	−0.007603	0.022*	
H28B	0.966591	0.594228	0.033405	0.022*	
C29	1.15445 (18)	0.68108 (14)	0.06399 (9)	0.0197 (3)	
H29A	1.170947	0.773751	0.058267	0.024*	
H29B	1.241895	0.639167	0.016749	0.024*	
C30	1.19716 (18)	0.61029 (13)	0.15383 (9)	0.0198 (3)	
H30A	1.324710	0.602970	0.151835	0.024*	
H30B	1.134237	0.666442	0.199967	0.024*	
C31	0.38822 (17)	0.41067 (13)	0.36585 (9)	0.0188 (3)	
H31A	0.345645	0.459203	0.417797	0.023*	
H31B	0.283955	0.397118	0.345283	0.023*	
C32	0.62358 (18)	0.28555 (14)	0.44600 (9)	0.0198 (3)	
H32A	0.659389	0.196276	0.477504	0.024*	
H32B	0.571759	0.345842	0.491046	0.024*	
C33	0.37437 (18)	0.18555 (13)	0.43519 (9)	0.0205 (3)	
H33A	0.449751	0.099609	0.450472	0.025*	
H33B	0.309815	0.173521	0.390086	0.025*	
C34	0.23937 (18)	0.21747 (14)	0.51833 (9)	0.0211 (3)	
H34A	0.301200	0.221478	0.566524	0.025*	
H34B	0.165479	0.305036	0.505587	0.025*	
C35	0.1226 (2)	0.11267 (15)	0.54839 (11)	0.0270 (3)	
H35A	0.066395	0.106049	0.498681	0.032*	
H35B	0.197237	0.026042	0.562874	0.032*	
C36	−0.0194 (2)	0.14142 (16)	0.62842 (12)	0.0321 (4)	
H36A	−0.091006	0.228698	0.615574	0.039*	
H36B	0.035182	0.140059	0.679530	0.039*	
H36C	−0.094333	0.074069	0.641870	0.039*	

### Atomic displacement parameters (Å^2^)

**Table:**

	*U*^11^	*U*^22^	*U*^33^	*U*^12^	*U*^13^	*U*^23^
N1	0.0183 (5)	0.0162 (5)	0.0162 (5)	−0.0042 (4)	−0.0001 (4)	−0.0030 (4)
N2	0.0177 (5)	0.0184 (6)	0.0202 (6)	−0.0048 (4)	−0.0022 (4)	−0.0021 (4)
N3	0.0188 (6)	0.0163 (5)	0.0176 (6)	−0.0054 (4)	−0.0021 (4)	0.0000 (4)
C4	0.0157 (6)	0.0183 (6)	0.0136 (6)	−0.0039 (5)	−0.0035 (5)	−0.0006 (5)
C5	0.0163 (6)	0.0163 (6)	0.0158 (6)	−0.0020 (5)	−0.0044 (5)	−0.0023 (5)
C6	0.0157 (6)	0.0184 (6)	0.0151 (6)	−0.0018 (5)	−0.0029 (5)	−0.0028 (5)
C7	0.0161 (6)	0.0192 (6)	0.0165 (6)	−0.0035 (5)	−0.0054 (5)	−0.0001 (5)
C8	0.0228 (7)	0.0151 (6)	0.0176 (6)	−0.0052 (5)	−0.0075 (5)	−0.0007 (5)
C9	0.0196 (6)	0.0177 (6)	0.0153 (6)	−0.0024 (5)	−0.0032 (5)	−0.0040 (5)
C10	0.0187 (6)	0.0162 (6)	0.0170 (6)	−0.0021 (5)	−0.0041 (5)	−0.0026 (5)
C11	0.0181 (6)	0.0182 (6)	0.0157 (6)	−0.0037 (5)	−0.0042 (5)	−0.0002 (5)
C12	0.0194 (6)	0.0185 (6)	0.0169 (6)	−0.0011 (5)	−0.0012 (5)	−0.0020 (5)
C13	0.0261 (7)	0.0159 (6)	0.0242 (7)	−0.0030 (5)	−0.0027 (6)	−0.0053 (5)
C14	0.0236 (7)	0.0148 (6)	0.0265 (7)	−0.0059 (5)	−0.0050 (6)	−0.0020 (5)
C15	0.0216 (7)	0.0195 (7)	0.0203 (7)	−0.0061 (5)	−0.0016 (5)	−0.0004 (5)
C16	0.0149 (6)	0.0210 (7)	0.0168 (6)	−0.0042 (5)	−0.0049 (5)	−0.0005 (5)
C17	0.0166 (6)	0.0181 (6)	0.0178 (6)	−0.0011 (5)	−0.0054 (5)	−0.0024 (5)
C18	0.0181 (6)	0.0175 (6)	0.0180 (6)	−0.0028 (5)	−0.0048 (5)	0.0004 (5)
C19	0.0178 (6)	0.0223 (7)	0.0152 (6)	−0.0027 (5)	−0.0054 (5)	−0.0005 (5)
C20	0.0185 (6)	0.0243 (7)	0.0167 (6)	−0.0019 (5)	−0.0042 (5)	−0.0054 (5)
C21	0.0201 (6)	0.0194 (7)	0.0201 (7)	−0.0033 (5)	−0.0061 (5)	−0.0043 (5)
C22	0.0157 (6)	0.0194 (6)	0.0198 (7)	−0.0027 (5)	−0.0044 (5)	−0.0026 (5)
C23	0.0146 (6)	0.0207 (7)	0.0164 (6)	−0.0037 (5)	−0.0032 (5)	−0.0022 (5)
C24	0.0197 (7)	0.0181 (7)	0.0235 (7)	−0.0027 (5)	−0.0034 (5)	−0.0003 (5)
C25	0.0217 (7)	0.0212 (7)	0.0262 (7)	0.0001 (5)	−0.0021 (6)	−0.0050 (6)
C26	0.0153 (6)	0.0298 (8)	0.0201 (7)	−0.0015 (5)	0.0003 (5)	−0.0033 (6)
C27	0.0162 (6)	0.0260 (7)	0.0209 (7)	−0.0070 (5)	−0.0021 (5)	0.0002 (5)
C28	0.0197 (7)	0.0211 (7)	0.0149 (6)	−0.0044 (5)	−0.0002 (5)	−0.0047 (5)
C29	0.0188 (6)	0.0195 (6)	0.0197 (7)	−0.0048 (5)	−0.0003 (5)	−0.0022 (5)
C30	0.0197 (6)	0.0189 (7)	0.0231 (7)	−0.0077 (5)	−0.0052 (5)	−0.0018 (5)
C31	0.0176 (6)	0.0185 (6)	0.0196 (7)	−0.0032 (5)	−0.0031 (5)	−0.0009 (5)
C32	0.0200 (7)	0.0220 (7)	0.0160 (6)	−0.0027 (5)	−0.0023 (5)	−0.0013 (5)
C33	0.0231 (7)	0.0166 (6)	0.0212 (7)	−0.0061 (5)	−0.0005 (5)	−0.0020 (5)
C34	0.0224 (7)	0.0183 (7)	0.0213 (7)	−0.0056 (5)	0.0005 (5)	−0.0018 (5)
C35	0.0272 (7)	0.0226 (7)	0.0292 (8)	−0.0096 (6)	0.0018 (6)	−0.0006 (6)
C36	0.0242 (8)	0.0263 (8)	0.0407 (9)	−0.0070 (6)	0.0067 (7)	−0.0031 (7)

### Geometric parameters (Å, º)

**Table:**

N1—C10	1.3875 (17)	C20—C21	1.3858 (19)
N1—C4	1.3891 (17)	C20—H20	0.9500
N1—C28	1.4542 (17)	C21—H21	0.9500
N2—C22	1.3895 (18)	C22—C27	1.3993 (19)
N2—C16	1.3925 (17)	C22—C23	1.4253 (19)
N2—C30	1.4630 (17)	C23—C24	1.3923 (19)
N3—C31	1.4684 (17)	C24—C25	1.386 (2)
N3—C33	1.4698 (17)	C24—H24	0.9500
N3—C32	1.4756 (17)	C25—C26	1.413 (2)
C4—C9	1.3986 (18)	C25—H25	0.9500
C4—C5	1.4098 (18)	C26—C27	1.376 (2)
C5—C6	1.4005 (18)	C26—H26	0.9500
C5—C11	1.4472 (18)	C27—H27	0.9500
C6—C7	1.3866 (19)	C28—C29	1.5286 (19)
C6—H6	0.9500	C28—H28A	0.9900
C7—C8	1.4115 (19)	C28—H28B	0.9900
C7—C31	1.5115 (18)	C29—C30	1.5347 (19)
C8—C9	1.3778 (19)	C29—H29A	0.9900
C8—H8	0.9500	C29—H29B	0.9900
C9—H9	0.9500	C30—H30A	0.9900
C10—C15	1.3988 (19)	C30—H30B	0.9900
C10—C11	1.4164 (18)	C31—H31A	0.9900
C11—C12	1.3884 (19)	C31—H31B	0.9900
C12—C13	1.3954 (19)	C32—H32A	0.9900
C12—H12	0.9500	C32—H32B	0.9900
C13—C14	1.416 (2)	C33—C34	1.5299 (19)
C13—H13	0.9500	C33—H33A	0.9900
C14—C15	1.381 (2)	C33—H33B	0.9900
C14—H14	0.9500	C34—C35	1.5271 (19)
C15—H15	0.9500	C34—H34A	0.9900
C16—C21	1.3948 (19)	C34—H34B	0.9900
C16—C17	1.4133 (19)	C35—C36	1.516 (2)
C17—C18	1.3980 (19)	C35—H35A	0.9900
C17—C23	1.4468 (18)	C35—H35B	0.9900
C18—C19	1.3902 (19)	C36—H36A	0.9800
C18—H18	0.9500	C36—H36B	0.9800
C19—C20	1.405 (2)	C36—H36C	0.9800
C19—C32	1.5179 (18)		
			
C10—N1—C4	108.38 (11)	C22—C23—C17	106.30 (12)
C10—N1—C28	126.84 (11)	C25—C24—C23	119.59 (13)
C4—N1—C28	124.78 (11)	C25—C24—H24	120.2
C22—N2—C16	108.10 (11)	C23—C24—H24	120.2
C22—N2—C30	127.04 (11)	C24—C25—C26	120.31 (13)
C16—N2—C30	124.72 (11)	C24—C25—H25	119.8
C31—N3—C33	111.29 (10)	C26—C25—H25	119.8
C31—N3—C32	114.56 (11)	C27—C26—C25	121.32 (13)
C33—N3—C32	113.55 (11)	C27—C26—H26	119.3
N1—C4—C9	129.49 (12)	C25—C26—H26	119.3
N1—C4—C5	109.42 (11)	C26—C27—C22	118.46 (13)
C9—C4—C5	121.02 (12)	C26—C27—H27	120.8
C6—C5—C4	119.43 (12)	C22—C27—H27	120.8
C6—C5—C11	133.58 (12)	N1—C28—C29	115.26 (11)
C4—C5—C11	106.48 (11)	N1—C28—H28A	108.5
C7—C6—C5	119.85 (12)	C29—C28—H28A	108.5
C7—C6—H6	120.1	N1—C28—H28B	108.5
C5—C6—H6	120.1	C29—C28—H28B	108.5
C6—C7—C8	119.25 (12)	H28A—C28—H28B	107.5
C6—C7—C31	121.17 (12)	C28—C29—C30	117.30 (11)
C8—C7—C31	119.55 (12)	C28—C29—H29A	108.0
C9—C8—C7	122.01 (12)	C30—C29—H29A	108.0
C9—C8—H8	119.0	C28—C29—H29B	108.0
C7—C8—H8	119.0	C30—C29—H29B	108.0
C8—C9—C4	118.03 (12)	H29A—C29—H29B	107.2
C8—C9—H9	121.0	N2—C30—C29	116.16 (11)
C4—C9—H9	121.0	N2—C30—H30A	108.2
N1—C10—C15	129.63 (12)	C29—C30—H30A	108.2
N1—C10—C11	109.09 (12)	N2—C30—H30B	108.2
C15—C10—C11	121.29 (12)	C29—C30—H30B	108.2
C12—C11—C10	119.92 (12)	H30A—C30—H30B	107.4
C12—C11—C5	133.47 (13)	N3—C31—C7	113.67 (11)
C10—C11—C5	106.59 (12)	N3—C31—H31A	108.8
C11—C12—C13	119.27 (13)	C7—C31—H31A	108.8
C11—C12—H12	120.4	N3—C31—H31B	108.8
C13—C12—H12	120.4	C7—C31—H31B	108.8
C12—C13—C14	120.02 (13)	H31A—C31—H31B	107.7
C12—C13—H13	120.0	N3—C32—C19	111.50 (11)
C14—C13—H13	120.0	N3—C32—H32A	109.3
C15—C14—C13	121.47 (13)	C19—C32—H32A	109.3
C15—C14—H14	119.3	N3—C32—H32B	109.3
C13—C14—H14	119.3	C19—C32—H32B	109.3
C14—C15—C10	118.00 (13)	H32A—C32—H32B	108.0
C14—C15—H15	121.0	N3—C33—C34	117.93 (11)
C10—C15—H15	121.0	N3—C33—H33A	107.8
N2—C16—C21	129.47 (13)	C34—C33—H33A	107.8
N2—C16—C17	109.55 (12)	N3—C33—H33B	107.8
C21—C16—C17	120.97 (12)	C34—C33—H33B	107.8
C18—C17—C16	119.87 (12)	H33A—C33—H33B	107.2
C18—C17—C23	133.27 (13)	C35—C34—C33	110.75 (12)
C16—C17—C23	106.64 (12)	C35—C34—H34A	109.5
C19—C18—C17	119.56 (13)	C33—C34—H34A	109.5
C19—C18—H18	120.2	C35—C34—H34B	109.5
C17—C18—H18	120.2	C33—C34—H34B	109.5
C18—C19—C20	118.91 (13)	H34A—C34—H34B	108.1
C18—C19—C32	120.73 (12)	C36—C35—C34	113.48 (13)
C20—C19—C32	119.96 (12)	C36—C35—H35A	108.9
C21—C20—C19	122.81 (13)	C34—C35—H35A	108.9
C21—C20—H20	118.6	C36—C35—H35B	108.9
C19—C20—H20	118.6	C34—C35—H35B	108.9
C20—C21—C16	117.33 (13)	H35A—C35—H35B	107.7
C20—C21—H21	121.3	C35—C36—H36A	109.5
C16—C21—H21	121.3	C35—C36—H36B	109.5
N2—C22—C27	129.80 (13)	H36A—C36—H36B	109.5
N2—C22—C23	109.31 (12)	C35—C36—H36C	109.5
C27—C22—C23	120.88 (13)	H36A—C36—H36C	109.5
C24—C23—C22	119.43 (12)	H36B—C36—H36C	109.5
C24—C23—C17	134.27 (13)		
			
C10—N1—C4—C9	−178.96 (13)	C23—C17—C18—C19	−175.18 (13)
C28—N1—C4—C9	0.4 (2)	C17—C18—C19—C20	−5.06 (19)
C10—N1—C4—C5	−2.13 (14)	C17—C18—C19—C32	167.69 (12)
C28—N1—C4—C5	177.18 (12)	C18—C19—C20—C21	6.4 (2)
N1—C4—C5—C6	−171.01 (11)	C32—C19—C20—C21	−166.42 (12)
C9—C4—C5—C6	6.13 (19)	C19—C20—C21—C16	−0.9 (2)
N1—C4—C5—C11	1.85 (14)	N2—C16—C21—C20	173.16 (13)
C9—C4—C5—C11	178.99 (12)	C17—C16—C21—C20	−5.76 (19)
C4—C5—C6—C7	−1.25 (19)	C16—N2—C22—C27	−177.87 (13)
C11—C5—C6—C7	−171.78 (13)	C30—N2—C22—C27	−2.0 (2)
C5—C6—C7—C8	−4.24 (19)	C16—N2—C22—C23	1.67 (14)
C5—C6—C7—C31	173.68 (11)	C30—N2—C22—C23	177.56 (12)
C6—C7—C8—C9	5.2 (2)	N2—C22—C23—C24	179.96 (12)
C31—C7—C8—C9	−172.75 (12)	C27—C22—C23—C24	−0.45 (19)
C7—C8—C9—C4	−0.45 (19)	N2—C22—C23—C17	0.28 (14)
N1—C4—C9—C8	171.28 (12)	C27—C22—C23—C17	179.87 (12)
C5—C4—C9—C8	−5.22 (19)	C18—C17—C23—C24	−7.3 (3)
C4—N1—C10—C15	−178.79 (13)	C16—C17—C23—C24	178.31 (14)
C28—N1—C10—C15	1.9 (2)	C18—C17—C23—C22	172.30 (14)
C4—N1—C10—C11	1.55 (14)	C16—C17—C23—C22	−2.08 (14)
C28—N1—C10—C11	−177.74 (12)	C22—C23—C24—C25	0.4 (2)
N1—C10—C11—C12	178.38 (12)	C17—C23—C24—C25	179.95 (14)
C15—C10—C11—C12	−1.3 (2)	C23—C24—C25—C26	−0.3 (2)
N1—C10—C11—C5	−0.40 (14)	C24—C25—C26—C27	0.2 (2)
C15—C10—C11—C5	179.91 (12)	C25—C26—C27—C22	−0.3 (2)
C6—C5—C11—C12	−8.0 (3)	N2—C22—C27—C26	179.90 (13)
C4—C5—C11—C12	−179.42 (14)	C23—C22—C27—C26	0.4 (2)
C6—C5—C11—C10	170.53 (14)	C10—N1—C28—C29	63.32 (17)
C4—C5—C11—C10	−0.87 (14)	C4—N1—C28—C29	−115.87 (14)
C10—C11—C12—C13	0.5 (2)	N1—C28—C29—C30	46.67 (17)
C5—C11—C12—C13	178.94 (14)	C22—N2—C30—C29	66.13 (17)
C11—C12—C13—C14	0.8 (2)	C16—N2—C30—C29	−118.63 (14)
C12—C13—C14—C15	−1.5 (2)	C28—C29—C30—N2	48.00 (17)
C13—C14—C15—C10	0.8 (2)	C33—N3—C31—C7	−156.62 (11)
N1—C10—C15—C14	−178.98 (13)	C32—N3—C31—C7	72.88 (14)
C11—C10—C15—C14	0.6 (2)	C6—C7—C31—N3	−134.49 (13)
C22—N2—C16—C21	177.94 (13)	C8—C7—C31—N3	43.42 (17)
C30—N2—C16—C21	1.9 (2)	C31—N3—C32—C19	−76.51 (14)
C22—N2—C16—C17	−3.04 (14)	C33—N3—C32—C19	154.11 (11)
C30—N2—C16—C17	−179.04 (11)	C18—C19—C32—N3	−69.92 (16)
N2—C16—C17—C18	−172.11 (11)	C20—C19—C32—N3	102.75 (14)
C21—C16—C17—C18	7.01 (19)	C31—N3—C33—C34	−59.87 (16)
N2—C16—C17—C23	3.17 (14)	C32—N3—C33—C34	71.15 (15)
C21—C16—C17—C23	−177.71 (12)	N3—C33—C34—C35	176.03 (12)
C16—C17—C18—C19	−1.39 (19)	C33—C34—C35—C36	−177.56 (13)

### Hydrogen-bond geometry (Å, º)

*Cg*1, *Cg*2, *Cg*3 and *Cg*4 are the centroids of the C22–C27, C4–C9, N2/C16/C17/C22/C23 and C10–C15 rings, respectively.

**Table:**

*D*—H···*A*	*D*—H	H···*A*	*D*···*A*	*D*—H···*A*
C13—H13···N3^i^	0.95	2.56	3.4858 (18)	166
C28—H28*A*···*Cg*1^ii^	0.99	2.72	3.4144 (15)	128
C30—H30*A*···*Cg*2^iii^	0.99	2.62	3.5224 (16)	152
C31—H31*B*···*Cg*3^iv^	0.99	2.83	3.7245 (15)	150
C36—H36*B*···*Cg*4^v^	0.98	2.99	3.9151 (18)	159

Symmetry codes: (i) *x*, *y*+1, *z*; (ii) −*x*+2, −*y*+1, −*z*; (iii) *x*+1, *y*, *z*; (iv) *x*−1, *y*, *z*+1; (v) −*x*+1, −*y*+1, −*z*+1.
